# Determinants of adherence to physical cancer rehabilitation guidelines among cancer patients and cancer centers: a cross-sectional observational study

**DOI:** 10.1007/s11764-020-00921-8

**Published:** 2020-09-28

**Authors:** Charlotte IJsbrandy, Petronella B. Ottevanger, Winald R. Gerritsen, Wim H. van Harten, Rosella P. M. G. Hermens

**Affiliations:** 1 Scientific Institute for Quality of Healthcare (IQ Healthcare), Radboud Institute for Health Science (RIHS), Radboud University Medical Center Nijmegen, PO Box 9101, Nijmegen, 6500 HB The Netherlands; 2 Department of Medical Oncology, Radboud Institute for Health Science (RIHS), Radboud University Medical Center Nijmegen, Nijmegen, The Netherlands; 3 Department of Radiation Oncology, Radboud Institute for Health Science (RIHS), Radboud University Medical Center, Nijmegen, The Netherlands; 4 Division of Psychosocial Research and Epidemiology, Netherlands Cancer Institute, Amsterdam, The Netherlands; 5 Department of Health Technology and Services Research, MB-HTSR, University of Twente, Enschede, The Netherlands

**Keywords:** Exercise, Health plan implementation, Guidelines, Neoplasm, Rehabilitation, Survivors

## Abstract

**Purpose:**

To tailor implementation strategies that maximize adherence to physical cancer rehabilitation (PCR) guidelines, greater knowledge concerning determinants of adherence to those guidelines is needed. To this end, we assessed the determinants of adherence to PCR guidelines in the patient and cancer center.

**Methods:**

We investigated adherence variation of PCR guideline-based indicators regarding [1] screening with the Distress Thermometer (DT), [2] information provision concerning physical activity (PA) and physical cancer rehabilitation programs (PCRPs), [3] advice to take part in PA and PCRPs, [4] referral to PCRPs, [5] participation in PCRPs, and [6] PA uptake (PAU) in nine cancer centers. Furthermore, we assessed patient and cancer center characteristics as possible determinants of adherence. Regression analyses were used to determine associations between guideline adherence and patient and cancer center characteristics. In these analyses, we assumed the patient (level 1) nested within the cancer center (level 2).

**Results:**

Nine hundred and ninety-nine patients diagnosed with cancer between January 2014 and June 2015 were included. Of the 999 patients included in the study, 468 (47%) received screening with the DT and 427 (44%) received information provision concerning PA and PCRPs. Subsequently, 550 (56%) patients were advised to take part in PA and PCRPs, which resulted in 174 (18%) official referrals. Ultimately, 280 (29%) patients participated in PCRPs, and 446 (45%) started PAU. Screening with the DT was significantly associated with information provision concerning PA and PCRPs (OR 1.99, 95% CI 1.47–2.71), advice to take part in PA and PCRPs (OR 1.79, 95% CI 1.31–2.45), referral to PCRPs (OR 1.81, 95% CI 1.18–2.78), participation in PCRPs (OR 2.04, 95% CI 1.43–2.91), and PAU (OR 1.69, 95% CI 1.25–2.29). Younger age, male gender, breast cancer as the tumor type, ≥2 cancer treatments, post-cancer treatment weight gain/loss, employment, and fatigue were determinants of guideline adherence. Less variation in scores of the indicators between the different cancer centers was found. This variation between centers was too low to detect any association between center characteristics with the indicators.

**Conclusions:**

The implementation of PCR guidelines is in need of improvement. We found determinants at the patient level associated with guideline-based PCR care.

**Implications for Cancer Survivors:**

Implementation strategies that deal with the determinants of adherence to PCR guidelines might improve the implementation of PCR guidelines and the quality of life of cancer survivors.

**Electronic supplementary material:**

The online version of this article (10.1007/s11764-020-00921-8) contains supplementary material, which is available to authorized users.

## Introduction

It is well known that the physical activity (PA) levels of patients affected by cancer generally decline [1], and only a small proportion of the patients with cancer get sufficient PA during treatment [2, 3]. The majority of patients fail to return to pre-diagnosis activity levels following treatment [2, 3]; however, PA improves both the physical and psychosocial functioning [4–15] of patients affected by cancer by decreasing fatigue [5, 7, 8, 16–25], improving cardiopulmonary fitness [7, 8, 16, 26], and improving quality of life (QoL) [8, 16, 19, 21, 26–32] while also decreasing cancer recurrence and cancer-specific mortality [33–36].

Evidence-based guidelines recommend the implementation of physical cancer rehabilitation programs (PCRPs) or other initiatives to improve the uptake of PA during and after cancer treatment [16, 35–43]. As the number of cancer survivors is still rising, the implementation of these guidelines has become an increasingly important topic worldwide [43, 44]. Depending on the cancer site and treatment, 30–90% of cancer patients require physical rehabilitation [45–48]. Regrettably, it appears that adherence to current guidelines on physical cancer rehabilitation (PCR) is low [49–53], and material on approaches to implementing PCR guidelines is scarce [54–58]. Patients who will accept and benefit from PCRPs can be identified by means of the Distress Thermometer (DT) [59, 60]. Using the DT for screening appears to be a good starting point for accomplishing adherence to current PCR guidelines, but evidence supporting this hypothesis is missing.

Most guidelines do not implement themselves and require implementation strategies [61, 62]. Various strategies have been advocated for the implementation of healthcare innovations, each based on different assumptions and theories on human behavior and organizations [61, 63–70]. Strategies tailored to determinants and barriers are recommended [71, 72] because tailoring is expected to contribute to their effectiveness [73] (odds ratios of 1.27 to 1.93 [74]). To design tailored implementation strategies, we used the stepwise theoretical framework of Grol and Wensing’s “Implementation of Change Model” [75, 76]. In doing so, we gained insight into current practice, potential determinants that predict adherence, and possible barriers and facilitators [77, 78] influencing PCR guideline implementation. Determinants and barriers often arise at multiple levels within the healthcare system (at the patient, healthcare provider (HCP), cancer center, and healthcare organization levels) [79]. To assess the barriers, we performed two earlier studies [77, 78]. We found multiple barriers at the level of PCR guidelines, PCRPs, and patients, but also at the level of HCPs, healthcare organization, and governance. Since a strategy that is additionally tailored to determinants of implementation is more effective, we also wanted to investigate determinants of PCR guideline implementation [73].

Research in other fields of care has demonstrated that a variety of determinants of the targeted patients and cancer centers can explain poor implementation of the recommended care [80–85]. Tailoring the strategy to these determinants will improve the chance to successful guideline implementation. Assessing determinants before starting the implementation is comparable with clinical practice in which a diagnosis is made so that the right treatment can be chosen [86]. However, determinants related to PCR guidelines being followed are currently not well known. To help tailor implementation strategies and maximize guideline adherence, and thereby the number of patients participating in PCRPs, more knowledge about the determinants of adherence to PCR guidelines is needed.

We aimed to (1) assess the adherence to PCR guidelines for patients with cancer. We hypothesized that the use of the distress thermometer (DT) could help to identify patients in need of PCRPs and persuade them to benefit from them; therefore, we also aimed to (2) assess the effect of the use of the DT on this adherence. Furthermore, we aimed to (3) analyze the determinants of adherence to PCR guidelines of (3a) cancer centers and (3b) the patients with cancer treated in these cancer centers.

## Methods

### Study design

An observational study was conducted to assess adherence to and determinants of PCR guidelines in nine cancer centers. This was done at cancer centers and on patients who have been treated in these cancer centers, while taking the clustering of data into account. The existing registration systems and patient and HCP questionnaires were utilized.

### Study population and recruitment

The patient cohort was recruited from the nine participating cancer centers located in categorical, university, teaching, and non-teaching hospitals in the Netherlands. The cancer registry was used for the selection of eligible patients: all patients with a history of breast, female organ, urogenital organ, gastrointestinal, and hematological malignancies diagnosed between January 2014 and June 2015 who had successfully completed their primary treatment without signs of recurrence or metastases. The treating physicians asked them whether they wanted to participate and give informed consent. One HCP at each center was asked to collect data on the characteristics of their cancer center.

### Data collection

Indicator scores for processes and patient outcomes of care as well as patient and center characteristics were measured to assess adherence to PCR guidelines and the determinants of guideline adherence. The indicators were based on (inter)national, evidence-based PCR guidelines [37, 87, 88]. A national panel of 10–12 professional experts and patients used the RAND-modified Delphi method to develop the indicators [89, 90]. We developed indicators that measure PCR guideline adherence that have the potential to be valuable, reliable, measurable, applicable, have improvement potential, have preferably minimum amount of missing data, and contain discriminatory capacity. The main indicator was distress screening with the DT [91, 92]. The other indicators were (1) information provision concerning PA and PCRPs, (2) advice to take part in PA and PCRPs, (3) referral to PCRPs, (4) participation in PCRPs, and (5) PA uptake (PAU). In supplement 1, the definitions of the psychometric characteristics used to develop and measure the quality of the indicators used is comprehensively explicated. All developed indicators showed to be valuable, reliable, measurable, applicable, have improvement potential, have minimum amount of missing data, and four indicators contain sufficient discriminatory capacity. Supplement 2 provides an overview of the range of potential values of each developed and measured indicator regarding the psychometric characteristics.

The indicators and patient characteristics were measured among patients by means of questionnaires. To assess cancer center characteristics, existing registry systems and questionnaires distributed among HCPs involved in cancer care in the nine cancer centers were used.

### Operational definition of the main indicator

#### Screening with the DT

The percentage is calculated by the number of patients included in the study who indicated in the patients’ questionnaire having received screening with the DT [91, 92] one or more times during their cancer treatment or follow-up visits from one or more healthcare professionals from the cancer center where they were treated for cancer, divided by the total number of patients included in the study who completed the patients’ questionnaire.

The questionnaire asked patients if they had received screening with the DT [91, 92]. A photograph of the DT was shown in the questionnaire.

Supplement 3 provides a detailed description of the DT and the other questionnaires used in the present study.

### Operational definition of the other indicators

#### Information provision concerning PA and PCRPs

The percentage is calculated by the number of patients who indicated in the patients’ questionnaire that they received information about PA and PCRPs from one or more healthcare professionals from the cancer center where they were treated for cancer one or more times during their cancer treatment or follow-up visits, divided by the total number of patients included in the study who completed the patients’ questionnaire.

#### Advice to take part in PA and PCRPs

The percentage is calculated by the number of patients who indicated in the patients’ questionnaire that they received advice to improve their PA and join a PCRP during and after cancer treatment from one or more healthcare professionals from the cancer center where they were treated for cancer one or more times during their cancer treatment or follow-up visits, divided by the total number of patients included in the study who completed the patients’ questionnaire.

#### Referral to PCRPs

The percentage is calculated by the number of patients who indicated in a patients’ questionnaire that they received a referral to a PCRP by one of their healthcare professionals from the cancer center where they were treated for the cancer one or more times during their cancer treatment or follow-up visits, divided by the total number of patients included in the study who completed the patients’ questionnaire.

#### Participation in PCRPs

The percentage is calculated by the number of patients who indicated in a patients’ questionnaire that they had joined a PCRP during and/or after their cancer treatment, divided by the total number of patients included in the study who completed the patients’ questionnaire.

#### PA uptake (PAU)

The percentage is calculated by the number of patients who indicated in a patients’ questionnaire that their PA had increased following cancer and cancer treatment compared with PA prior to cancer treatment, divided by the total number of patients included in the study who completed the patients’ questionnaire.

### Characteristics of patients and cancer centers

#### Patient characteristics

The patient characteristics were age (continuous), gender (male or female), nationality (Dutch or other nationality), tumor type (breast, female organ, urogenital organ, and gastrointestinal and hematological malignancies), type of any treatment previously received for treating the tumor (surgery, chemotherapy, radiotherapy, hormonal therapy, or other), multi-treatment (≥2/<2 cancer treatments), comorbidities (≥2/<2), post-cancer treatment weight gain/loss (gain, stable, loss), residential circumstances (alone or cohabitating), educational level (high, middle, or low), and employment status (employed or unemployed).

Moreover, patient-reported outcomes (PROs) were included in patient characteristics.

### Patient-reported outcomes (PROs)

#### QoL

The European Organization for Research and Treatment of Cancer Quality of Life Questionnaire (EORTC QLQ-C30) [93] was used to measure the QoL of the patients. A measurement model for the QLQ-C30 that yields a single summary score based on 13 scales (27 items) was also calculated [94].

#### Fatigue

The Multidimensional Fatigue Inventory-20 (MFI-20) questionnaire [95, 96] was used to measure patient fatigue.

#### Patient empowerment

Patient empowerment was defined as the individual knowledge, skills, and confidence for managing the patient’s own health and healthcare. The state of patient empowerment was measured using the patient activity measurement-13 (PAM-13) [97, 98].

#### Cancer center characteristics

The cancer center characteristics were type of hospital (categorical, university, teaching, and non-teaching), Multidisciplinary Oncological Rehabilitation Board (MORB) available (yes or no), standardized screening with DT (yes or no), and PCRP in cancer center or connected cancer center available (internally, externally, or not at all). A MORB is a group of HCPs involved in oncological rehabilitation (e.g., surgeons, radiotherapists, medical oncologists, gynecologists, urologists, rehabilitation physicians, sports-medicine physicians, physiotherapists, physician assistants, nurses, and psychologists) interacting dynamically, interdependently, and adaptively toward common, valued rehabilitation plans for the patients.

### Data analysis

We used the Statistical Package for the Social Sciences (IBM SPSS Statistics version 22 for Windows; SPSS, Chicago, IL, USA) to enter the collected data in a database. We used descriptive analyses (frequencies, percentages, means, and SD or median and interquartile ranges) to describe patient characteristics and the scores for adherence to the indicators.

To determine whether our data were normally distributed, we examined the distribution of our continuous outcome measures and carried out quantile–quantile (Q-Q) plots. We also calculated the skewness and kurtosis of these variables. For all variables, both values of the skewness and kurtosis were between − 1 and + 1, and Q-Q plots showed a straight line; therefore, they met normality requirements.

Because of the hierarchical structure of the study (patients nested within cancer centers), we performed multilevel analyses. We used univariate multilevel analyses for the indicators; the indicators were used as dependent variables. The characteristics of the patients and cancer centers were used as independent variables. Variables with *P* < 0.20 in the univariate multilevel analysis were selected for the multilevel multivariate analysis. Collinearity among independent variables was tested with either a Pearson or Spearman correlation. If two independent variables (rho >0.6) correlated strongly, only the most clinically relevant characteristic was included. Multicollinearity was tested with the variance inflation factor, with values greater than 10 indicating multicollinearity.

We wanted to assess the extent to which the indicator scores could be explained by characteristics of (1) cancer centers, but also (2) the patients who were treated in these cancer centers, while taking the clustering of data into account. Our dataset contains information at the patient level from nine different cancer centers for each indicator.

For this purpose, we used SAS software (SAS 9.2 for Windows; SAS Institute, Cary, North Carolina, USA) for our multilevel multivariable regression analyses. We used the Glimmix procedure for dichotomous data and the MIXED procedure for continuous data to determine (1) the association between the scores of the indicators and patient and cancer center characteristics, and (2) the association between proper screening and the other indicators. The other indicators were information provision concerning PA and PCRPs, advice to take part in PA and PCRPs, referral to PCRPs, participation in PCRPs and PAU. The patient characteristics were included in the model as confounders in the analysis.

Multilevel models were used because these models take into consideration the variability associated with each level of nesting and the within-patient correlation. In these analyses, it was assumed that the patient (level 1) nested within the cancer center (level 2). We ran a model with a random intercept and all other variables fixed. Significance for multivariate analyses was set at *P* < 0.05, based on two-sided testing. Odds ratios (OR) and 95% confidence intervals (95% CI) were used to describe (1) the association between the scores of the indicators and patient and cancer center characteristics, and (2) the association of proper screening and the other indicators.

The intra-class coefficient (ICC) was calculated for each outcome to obtain insight into the clustering effect of the cancer centers.

## Results

Nine cancer centers and their patients were recruited and included in the study. Of the 2069 patients who matched the inclusion criteria invited, 1211 patients (59%) responded, and 999 patients (48%) agreed to participate and gave informed consent.

### Patient and cancer center characteristics

The mean age of the participants was 66.3 years; 60.7% were female and 94% had a Dutch background. The participants had a history of cancer of the breast (31.1%), female organs (16.0%), urogenital organs (23.6%), gastrointestinal (27.7%), and hematological malignancies (1.5%). Eighty-five percent had undergone surgery, 39.8% received chemotherapy, 42.1% radiotherapy, and 21.7% hormonal therapy. Fifty-nine percent had received two or more cancer treatments and 31.7% had two or more comorbidities. Post-cancer treatment, 36.4% gained and 13.2% lost weight, while 50.4% kept a stable weight. Of the participants, 80.5% were cohabiting. As level of education, 38.3% of patients have finished low, 40.4% middle, and 21.2% a higher level of education and 25% were employed. The mean Global Health Status score was 77.5 (SD 18.0), the mean physical function score was 82.5 (SD 18.3), and the mean EORTC-QLQ-C30 summary score was 40.8 (SD 5.4). The Mean General Fatigue score was 10 (SD 4.6) and the Mean Physical Fatigue score was 9.6 (SD 4.4). The mean PAM-13 Total Score was 55.9 (SD 13.1).

In the study, one categorical, two university, two teaching, and four non-teaching hospitals participated related to respectively 5.5, 20.5, 25.2, and 48.7% of the accrued patients. One center had a MORB available. Five hospitals performed standardized screening with the DT. Eight hospitals delivered a PCRP, of which four hospitals delivered internally and four hospitals externally.

Table 1 outlines the characteristics of the patients treated for the various types of cancer. Table 2 outlines the characteristics of the nine cancer centers.

**Table 1:** Characteristics of the patients

	**Total**	**Screened with DT**	**Not screened with DT**
Number of patients	999	468	524
Characteristics			
Age in years			
Mean (SD)	66.3 (11.4)	63.1 (11.6)	69.0 (10.4)
	**Total**	**Screened with DT**	**Not screened with DT**
	Number of patients (%*)	Number of patients (%*)	Number of patients (%*)
Female gender	595 (60.7)	345 (74.5)	248 (48.4)
Dutch nationality	913 (93.5)	429 (93.4)	480 (93.4)
Tumor type			
Breast	311 (31.1)	207 (44.2)	103 (19.7)
Female organs	160 (16.0)	81 (17.3)	78 (14.9)
Urogenital organs	236 (23.6)	66 (14.1)	169 (32.3)
Gastrointestinal	277 (27.7)	104 (22.2)	169 (32.3)
Hematological malignancies	15 (1.5)	10 (2.1)	5 (1.0)
Previously received treatment			
Surgery	844 (84.5)	421 (90.0)	417 (79.6)
Chemotherapy	398 (39.8)	235 (50.2)	159 (30.3)
Radiotherapy	421 (42.1)	228 (48.7)	191 (36.5)
Hormonal therapy	217 (21.7)	133 (28.4)	83 (15.8)
Other	53 (5.3)	20 (4.3)	33 (6.3)
≥ 2 cancer treatments	590 (59.1)	328 (70.1)	258 (49.2)
≥ 2 comorbidities	317 (31.7)	167 (35.7)	149 (28.4)
Post-cancer treatment weight gain/loss			
Gain	353 (36.4)	193 (42.1)	157 (31.0)
Stable	489 (50.4)	194 (42.4)	294 (58.0)
Loss	128 (13.2)	71 (15.5)	56 (11.0)
Cohabiting	799 (80.5)	380 (81.9)	416 (79.7)
Educational level			
Low	379 (38.3)	169 (36.6)	205 (39.4)
Middle	400 (40.4)	197 (42.6)	201 (38.7)
High	210 (21.2)	96 (20.8)	114 (21.9)
Employed	246 (25.3)	141(30.9)	104 (20.4)
Department type			
Categorical	55 (5.5)	27 (5.8)	27 (5.2)
University	205 (20.5)	76 (16.2)	129 (24.6)
Teaching	252 (25.2)	108 (23.1)	142 (27.1)
Non-teaching	487 (48.7)	257 (54.9)	226 (43.1)
EORTC-QLQ-C30			
Global Health Status/QoL (SD)	77.5 (18.0)	77.5 (17.4)	77.5 (18.5)
Physical function (SD)	82.5 (18.3)	82.1 (17.9)	82.9 (18.8)
Mean summary score (SD)	40.8 (5.4)	40.7 (5.4)	40.8 (5.4)
MFI-20 score			
Mean general fatigue (SD)	10.0 (4.6)	10.5 (4.5)	9.6 (4.6)
Mean physical fatigue (SD)	9.6 (4.4)	9.9 (4.3)	9.3 (4.4)
PAM-13			
Mean total score (SD)	55.9 (13.1)	56.4 (12.5)	55.6 (13.7)

DT, Distress Thermometer; EORTC QLQ-C30, The European Organization for Research and Treatment of Cancer Quality of Life Questionnaire; MFI-20, Multidimensional Fatigue Inventory-20; PAM-13, Patient Activity Measurement-13

*valid percentage

**Table 2:** Characteristics of the cancer centers

	Number of facilities	Center 1	Center 2	Center 3	Center 4	Center 5	Center 6	Center 7	Center 8	Center 9
Total number of cancer centers	9									
Type of hospital										
Categorical	1						***√***			
University	2	***√***						***√***		
Teaching	2			***√***					***√***	
Non-teaching	4		***√***		***√***	***√***				***√***
MORB available	1						**√**			
Standardized screening with DT	5		**√**	**√**		**√**	**√**		**√**	
PCRP in cancer center										
Internally	4			**√**			**√**		**√**	**√**
Externally	4	**√**	**√**			**√**		**√**		

DT, Distress Thermometer; MORB, Multidisciplinary Oncological Rehabilitation Board; PCRP, physical cancer rehabilitation program

#### Indicator adherence

The score of screening with the DT was 47.2%. Information provision concerning PA and PCRPs scored 44.1%, advice to take part in PA and PCRPs scored 55.6%, referral to PCRPs scored 17.7%, participation in PCRPs scored 28.6%, and PAU scored 45.3%. The indicator scores were higher for the patients who were screened with the DT. Information provision concerning PA and PCRPs scored 55.7% vs. 33.5%, advice to take part in PA and PCRPs 67.0% vs. 45.3%, referral to PCRPs 24.7% vs. 11.5%, participation in PCRPs 38.1% vs. 20.2%, and PAU 54.0% vs. 37.3% for respectively patients screened with the DT versus patients not screened with the DT. Screening with the DT was significantly associated with improved information provision concerning PA and PCRPs (OR 1.99, 95% CI 1.47–2.71), advice to take part in PA and PCRPs (OR 1.79, 95% CI 1.31–2.45), referral to PCRPs (OR 1.81, 95% CI 1.18–2.78), participation in PCRPs (OR 2.04, 95% CI 1.43–2.91), and PAU (OR 1.69, 95% CI 1.25–2.29). Table 3 shows the effect of screening with the DT on the other indicators.

**Table 3:** Effect of screening with Distress Thermometer on other indicator scores in the multilevel analysis

Effect of screening with Distress Thermometer on	Number of patients	Uncorrected			Corrected for confounders**		
		OR	95% CI	*P* value*	OR	95% CI	*P* value*
Information provision concerning PA and PCRPs	856	2.28	1.72 to 3.01	< 0.0001	1.99	1.47 to 2.71	< 0.0001
Advice to take part in PA and PCRPs	868	2.33	1.76 to 3.08	< 0.0001	1.79	1.31 to 2.45	0.0003
Referral to PCRPs	866	2.61	1.80 to 3.78	< 0.0001	1.81	1.18 to 2.78	0.0067
PCRP participation	865	2.64	1.93 to 3.61	< 0.0001	2.04	1.43 to 2.91	< 0.0001
PAU	867	2.16	1.64 to 2.84	< 0.0001	1.69	1.25 to 2.29	0.0007

CI, confidence interval; OR, odds ratio; PA, physical activity;  PCRP, physical cancer rehabilitation program; PAU, physical activity uptake

*The patient characteristics age, gender, comorbidities (≥ 2/< 2), tumor type, multi-treatment (≥ 2/< 2), weight change after cancer treatment, work status, and the outcome of the Multidimensional Fatigue Inventory-20 were included in the model as confounders in the multilevel analysis

### Determinant analysis

The indicator for *screening with the DT* scored significantly higher with the determinants younger age, female gender, breast cancer as type of tumor, two or more cancer treatments, and post-cancer treatment weight gain/loss.

The determinants younger age, male gender, and breast cancer as type of tumor resulted in significant higher scores of *information provision concerning PA and PCRPs*.

The determinants two or more cancer treatments, post-cancer treatment weight gain, employment, and higher MFI-20 mean general fatigue scores resulted in significant higher scores of *advice to take part in PA and PCRPs*.

The determinants younger age, male gender, breast cancer as type of tumor, two or more cancer treatments, post-cancer treatment weight gain, and higher MFI-20 mean general fatigue scores resulted in significant higher scores of *referral to PCRPs*.

The determinants younger age, male gender, breast cancer as type of tumor, post-cancer treatment weight gain, and higher MFI-20 mean general fatigue scores resulted in significant higher scores of *participation in PCRPs.*

The determinants younger age and post-cancer treatment weight gain/loss resulted in higher scores of *PAU*.

Table 4 shows multilevel associations of patient characteristics with the measured indicators.

**Table 4:** Patient characteristics and their association with the indicator scores in the multilevel analysis

**Received screening with Distress Thermometer**	**Number of patients**	**OR**	**95% CI**	***P*** value*
	992			
Age		0.96	0.95 to 0.98	< 0.0001
Male		0.57	0.36 to 0.91	0.0194
Tumor type				0.0011
Breast		1.00		
Female organs		0.39	0.21 to 0.73	0.0031
Urogenital organs		0.30	0.15 to 0.63	0.0014
Gastrointestinal		0.46	0.28 to 0.75	0.0019
≥2 cancer treatments		1.44	1.04 to 2.00	0.0300
Post-cancer treatment weight gain/loss				0.0245
Stable		1.00		
Gain		1.46	1.06 to 2.01	0.0222
Loss		1.63	1.04 to 2.54	0.0316
**Received information provision concerning PA and PCRPs**	**Number of patients**	**OR**	**95% CI**	***P*** value*
	968			
Age		0.97	0.96 to 0.98	< 0.0001
Male		1.63	1.04 to 2.56	0.0344
Tumor type				< 0.0001
Breast		1.00		
Female organs		0.40	0.26 to 0.60	< 0.0001
Urogenital organs		0.29	0.17 to 0.51	< 0.0001
**Received advice to take part in PA and PCRPs**	**Number of patients**	**OR**	**95% CI**	***P*** value*
	989			
≥2 cancer treatments		2.01	1.48 to 2.72	< 0.0001
Post-cancer treatment weight gain/loss				< 0.0001
Stable		1.00		
Gain		2.07	1.50 to 2.85	< 0.0001
Employed		2.00	1.42 to 2.80	< 0.0001
MFI-20—mean general fatigue score		1.08	1.05 to 1.12	< 0.0001
**Received referral to PCRPs**	**Number of patients**	**OR**	**95% CI**	***P*** value*
	982			
Age		0.98	0.96 to 1.00	0.0137
Male		3.26	1.56 to 6.82	0.0018
Tumor type				0.0020
Breast		1.00		
Female organs		0.33	0.14 to 0.77	0.0107
Urogenital organs		0.13	0.04 to 0.37	0.0002
Gastrointestinal		0.31	0.15 to 0.65	0.0020
≥2 cancer treatments		2.25	1.36 to 3.73	0.0016
Post-cancer treatment weight gain/loss				0.0011
Stable		1.00		
Gain		2.11	1.39 to 3.20	0.0005
MFI-20—mean general fatigue score		1.07	1.02 to 1.11	0.0027
**Participated in PCRPs**	**Number of patients**	**OR**	**95% CI**	***P*** value*
	978			
Age		0.98	0.96 to 1.00	0.0009
Male		1.92	1.08 to 3.41	0.0265
Tumor type				0.0003
Breast		1.00		
Female organs		0.38	0.21 to 0.69	0.0017
Urogenital organs		0.22	0.10 to 0.48	0.0001
Gastrointestinal		0.34	0.19 to 0.60	0.0002
Post-cancer treatment weight gain/loss				< 0.0001
Stable		1.00		
Gain		2.08	1.47 to 2.94	< 0.0001
MFI-20– mean general fatigue score		1.10	1.06 to 1.14	< 0.0001
**Increase in PAU**	**Number of patients**	**OR**	**95% CI**	***P*** value*
	992			
Age		0.97	0.95 to 0.98	< 0.0001
Post-cancer treatment weight gain/loss				0.0002
Stable		1.00		
Gain		1.60	1.20 to 2.14	0.0016
Loss		2.12	1.40 to 3.20	0.0004

CI, confidence interval; OR, odds ratio; MFI, Multidimensional Fatigue Inventory-20; PA, physical activity; PCRP, physical cancer rehabilitation program; PAU, physical activity uptake

*Valid percentage

Univariate and multivariable multilevel regression analyses were performed but showed less variation in scores of the indicators between the different cancer centers. The ICCs of the outcomes varied between 0 and 0.085. This means that maximum 8.5% of the variation in an indicator could be explained by differences between cancer centers. This variation between centers was too low to detect any association between center characteristics (such as type of hospital, availability of a MORB, existence of standardized DT screening, and existence of PCRPs) with the indicators.

## Discussion

In this observational study, we investigated the adherence to PCR guideline-based indicators and analyzed the associated determinants. We found less than 50% adherence for indicators on screening with the DT, information provision concerning PA and PCRPs, referral to PCRPs, participation in PCRPs, and PAU. Only the indicator for advice to take part in PA and PCRPs scored higher than 50%. Screening with the DT was significantly associated with higher scores of all other indicators. Younger age, male gender, breast cancer as type of tumor, two or more cancer treatments, post-cancer treatment weight gain/loss, employment, and higher scores on MFI-20 mean general fatigue score were positively associated with higher indicator scores. The variation in center characteristics was too low to detect any association with the indicators.

Knowledge and understanding of the determinants of adherence to evidence-based PCR guideline-based indicators in the present study, together with previously published studies assessing the barriers of adherence to evidence-based PCR guidelines [77, 78], can assist HCPs in developing tailored strategies which can lead to improved adherence to PCR guidelines [75] by considering current practice as well as determinants of and barriers to adherence.

### Screening with the DT

Screening is a key aspect in the delivery of healthcare. Patients who will accept and benefit from rehabilitation programs can be identified and encouraged to participate in PCRPs by using the DT [59, 60]. Encouraging screening with the DT in daily cancer care will help promote the implementation of PCR guidelines. In our study, 47% of the patients were screened with the DT. Other studies found comparable percentages of screening with the DT of 40–50% [99]. The score of screening with the DT shows room for improvement, especially because the screening was significantly positively associated with higher scores of the other indicators, with ORs between 1.69 and 2.04. Therefore, encouraging screening with the DT is a good first step toward improving adherence to the current PCR guidelines.

### Determinants

This is one of the first studies to investigate determinants at the patient and cancer center levels for PCR guideline-based indicators. Other studies examining determinants of guideline adherence have been carried out in several other areas of cancer care, including treatment guidelines for lung, prostate, and gastrointestinal cancers. They found low guideline adherence rates and differences in delivered care associated with hospital type and patient age, gender, and disease stage [100–103] as well as educational level [104, 105] and employment status [106]. In addition, implementation strategies developed with knowledge of determinants in these other areas of cancer care did achieve improvement of guideline adherence. Therefore, knowledge of determinants is useful in creating tailored strategies for implementing PCR guidelines.

We detected a higher screening score for women, but higher scores for information provision, referral, and participation in PCRPs for men. Gender disparity in the use of cancer rehabilitation care and other healthcare services has been noted before in the literature [83, 107–109].

Traditional notions of masculinity that emphasize the values of being autonomous and less emotional may lead men to be reluctant to express emotion or distress [110–112]. In addition to the higher levels of media attention being paid to the post-cancer physical and psychosocial symptoms experienced by women, HCPs might be influenced by gender bias and less aware of screening men for distress. Men also seem to be more eager for sufficient explanations concerning screening in order to make a decision to participate [56, 57].

Besides screening, gender bias has been reported to affect HCP referral and treatment decisions and may also influence decisions on advising women for increasing PA or referral to PCRPs [113–115]. Women’s gender-specific roles and PA preferences may also contribute to women not participating in PCRPs. Contemporary the burden of cancer is evenly distributed between the different sexes. Currently, one in five men and one in six women will be diagnosed with cancer [116]; therefore, attention should be paid to improve screening of males, and improving information provision and referrals to PCRPs for female survivors of cancer. We did not distinguish our strategies on sex in our research. Future research can be used to differentiate which strategies are more effective for men and women.

We also found tumor type to be a determinant. Patients with breast cancer receive more screening, information, and referral to PCRPs, and they participate more in PCRPs. Indicator scores were lower for patients with a history of female organ, urogenital organ, and gastrointestinal malignancies. One reason for this is that most initiatives for improving PCR guideline adherence are designed for and focused on breast cancer. Screening of patients with breast cancer with the DT was relatively well adhered to, as not screening means no accreditation for breast cancer care as required by the patient organization for patients with breast cancer. Patients with gastrointestinal and female organ malignancies judge their cancer care to be of lower quality than that of patients with other tumor types [117]. After completing their primary treatment, patients with gastrointestinal malignancies also rated the information provided as significantly lower in quality than that of patients with breast cancer [118]. Worldwide, malignancies of the gastrointestinal, reproductive, and urogenital systems account for approximately 35% of all malignancies, which is three times the incidence of breast cancer [119, 120]. Therefore, it might be beneficial to preferably focus the strategy on cancer patients and their HCPs in the care pathways for gastrointestinal, female organ, and urogenital organ oncology.

The recruitment of patients with abdominopelvic cavity tumors to PCRPs is difficult [7, 8, 26, 121]. HCPs are more hesitant to refer patients who have undergone major abdominal surgery to PCRPs and typically advise patients to refrain from PA for a number of weeks after surgery [7]. Teaching the HCPs about the positive associations of PA with less physical and psychosocial symptoms and even improved mortality [33, 34, 122–124] might be a good strategy. In addition, tailored PA guidelines need to be developed since these patients require different PCRPs due to a different range of morbidities and needs. The introduction of accreditation for PCR guideline-based care that has proved successful for patients with breast cancer into the pathways for gastrointestinal, female organ, and urogenital organ oncology might be another strategy.

Two or more cancer treatments showed to be a determinant. Patient with fewer treatments overall have fewer visits to the cancer center and encounter fewer HCPs who provide them PCR guideline-based care. For all treatment modalities, it should be clear when, who, and where the PCR care is delivered, preferably stated in a treatment protocol. PCRPs delivered through practical avenues such as print materials, telephone counseling, and web-based programs are an alternative [125–129] for patients with fewer visits to the cancer center. Web-based PCRPs with online encouragement, online diaries, and online physical activity programs proved to be feasible with median vigorous PAU over time, and the burden for HCPs appeared to be limited [130–132].

Patients with post-cancer treatment weight gain/loss had better adherence to PCR guideline-based indicators. Cancer patients experience weight changes due to the cancer itself or to the cancer treatments, such as loss of muscular mass and increased fat mass [133–136]. The weight change might be the reason for paying more attention to PCR. A referral to a dietician might more readily lead patients to PCRPs as a means of returning to their old weight. In addition, PA is one of the main treatments for weight imbalance since it reduces fat mass and improves muscle mass and has a potential role in preventing and treating cachexia [136, 137].

The ORs of age being 0.96–0.98 and ORs of the MFI-20 mean general score being 1.07–1.10 are numerically very close to an OR of 1.00. The absolute influence of the determinants age and fatigue (the MFI-20 mean general score) on the indicator scores will therefore be negligible and not clinically relevant.

However, a higher age has previously been found to be associated with negative patterns in delivered care and lower levels of PA [138–142]. In addition, most cancer patients have higher fatigue scores [95], and fatigue is a common and debilitating side effect of cancer and its treatment [143]. It is known that PA can reduce fatigue after the treatment of cancer [144]. More research is necessary to explore the additional effect of strategies focusing on patients with fatigue and of a higher age.

### Strengths and limitations

Our study has several strengths. One is that we thoroughly followed the RAND-modified Delphi method [89, 90], which led to the discovery of valid indicators which formed an important basis for measuring guideline-based PCR care. Another is the large study sample of 999 patients, which might have contributed to a reliable dataset for the investigation of the adherence and the analysis of the determinants associated with optimal PCR care.

There are also some limitations which need to be addressed; for example, possible selection bias. Only patients of cancer centers who were willing to participate in our study were included. One can assume these patients have better adherence to PCR guideline-based indicators since these centers are more dedicated to improving this aspect of cancer care. Thus, we expect lower indicator scores in centers less committed to achieving this goal. Further research should also include patients from cancer centers not motivated to implement PCRPs.

One could expect that also organizational characteristics would be associated with performance on indicators related to the provision of distress screening and rehabilitation programs to cancer survivors. Univariate and multivariable multilevel regression analyses showed less variation in scores of the indicators between the different cancer centers. The ICC is calculated as the ratio of the between variance and the total variance (between and within variance). The ICC gives information of the degree of correlation among patients within a cancer center and the proportion of total variance that is attributed to the cluster level (cancer centers). The ICCs of the outcomes varied between 0 and 0.085. This means that maximum 8.5% of the variation in an indicator could be explained by differences between cancer centers, predicting a low chance of between-cluster variability. This variation between centers was too low to detect any association between center characteristics with the indicators. This might be caused by the limited sample size of nine cancer centers and the variation in characteristics between them, indicating that participation of more centers with more variation in characteristics is needed in future research to analyze cancer centers’ characteristics associated with the indicators.

Possible determinants influencing PCR guideline implementation often arise at multiple levels in the healthcare system (patient, HCPs, cancer center, and healthcare organization levels). Currently, cancer care is frequently provided by a multidisciplinary team of HCPs situated in a cancer center. This results in interactive, coordinated care; therefore, we only explored determinants of the cancer centers. However, there may be compelling reasons for both lack of adherence and adherence due to determinants of the individual HCPs, particularly because HCPs’ limited knowledge and skill levels, negative approach, non-commitment to PCRPs, difference in attitude about timing and strategies for cancer rehabilitation, and fear of additional workload all hinder proper PCR care [77, 78, 145, 146]. On the level of the referring providers, limited knowledge levels concerning PCRPs and PCR guidelines hinder proper screening of patients. Moreover, lack of knowledge and skills among HCPs resulted in a lack of qualified information provision for the patients. It also resulted in a lack of guidance in finding the right PCRP and a successful referral for joining the PCRP, both being barriers that impede proper PCR care [77, 78, 145, 146].

## Conclusions

Our study highlights the need for improvement in the implementation of PCR guidelines. It demonstrates that there are numerous determinants at the patient level associated with PCR guideline-based indicators. To ascertain cancer center determinants, more cancer centers with greater variation in characteristics are needed in future research. We discovered that screening with the DT is significantly positively associated with higher indicator scores and should be the first step in any successful implementation. The next step is developing and evaluating an implementation strategy based on knowledge of the determinants.

## Electronic supplementary material

ESM 1 (DOCX 28.9 kb).

ESM 2 (DOCX 18.7 kb).

ESM 3 (DOCX 30.8 kb).

## Data Availability

All data generated or analyzed in the present study are included in this published article and its supplementary information files.

## Abbreviations

EORTC QLQ-C30: The European Organization for Research and Treatment of Cancer Quality of Life Questionnaire
ICC: Intra-class coefficient
MFI-20: Multidimensional Fatigue Inventory-20
OR: Odds ratio
PA: Physical activity
PAM-13: Patient Activity Measurement-13
PAU: Physical activity uptake
PCR: Physical cancer rehabilitation
PCR guideline: Physical cancer rehabilitation guideline
PCRP: Physical cancer rehabilitation program
QoL: Quality of life
