# Supplementary material for: Determinants of adherence to physical cancer rehabilitation guidelines among cancer patients and cancer centers: a cross-sectional observational study
Source: J Cancer Surviv. 2020 Sep 28;15(1):163–77. doi: 10.1007/s11764-020-00921-8 (PMC7822788; doi:10.1007/s11764-020-00921-8)
Supplement: Supplementary file 1 — (DOCX 28.9 kb). [file 11764_2020_921_MOESM1_ESM.docx]

**Supplement 1 definitions of the psychometric characteristics used to measure the quality of the indicators used for physical cancer rehabilitation (PCR) guideline adherence.**

We developed indicators that measure physical cancer rehabilitation (PCR) guideline adherence that have the potential to be valuable, reliable, measurable, applicable, have improvement potential, have preferably minimum amount of missing data and contain discriminatory capacity.

As we thoroughly followed the steps of a RAND-modified Delphi method[^1^](#_ENREF_1)^,^[^2^](#_ENREF_2) the indicators form an important basis to measure adherence to guideline-based PCR care. The RAND-modified Delphi[^3^](#_ENREF_3) facilitates a systematic process of creating quality indicators and combines a multidisciplinary panel with evidenced-based information. It is a frequently used method that has proven to result in valid, reliable, measurable, applicable and achievable quality indicators[^4^](#_ENREF_4)^,^[^5^](#_ENREF_5)

- ***Validity:*** in this study validity is subdivided into face and content validity:
  - Face validity is defined as the degree to which an indicator seems to measure what it reports to measure.
  - Content validity is the extent to which the elements composing the indicator are relevant and representative of the construct they want to measure.

To ensure face and content validity, the participants of our Rand-modified Delphi group included healthcare providers that developed the Dutch PCR guideline.

- ***Reliability:*** reliability is usually defined in terms of measurement reproducibility. If the measurement of indicator data by two different data-collectors is highly comparable, there is reliability[^6^](#_ENREF_6).

We expect a high reliability of the indicators. We extracted the data from an patients’ questionnaire.

- ***Measurability:*** an indicator is considered measurable if data to fill the numerator and denominator of the indicator can be made available through data collection (e.g. medical records, complication or treatment databases or a survey).

We expect a high measurability of the indicators. We measured the data though an patients’ questionnaire.

- ***Applicability:*** for accurate performance assessment, an indicator is preferably applicable to a substantial proportion of patients; this is referred to as applicability[^7^](#_ENREF_7). Preferably, an indicator is applicable to at least 10% of the population.

The developed indicators used in this study are applicable to 100% of the population. We only selected patients with a history of breast, female organs, urogenital organs, gastrointestinal and hematological malignancies who had successfully passed their primary treatment without signs of recurrence or metastases.

- ***Improvement potential:*** when an indicator is used to detect changes in clinical performance, it is a prerequisite that improvement is possible at all; if overall performance for a certain indicator is already very high, the indicator has no improvement potential. We defined improvement potential as an indicator score <90%[^8^](#_ENREF_8).

All indicators had improvement potential. The percentages of adherence for the indicators were between 28.6%-55.6%.

- ***Missing data:*** calculating reliable indicator scores starts with the use of reliable data. Missing data may bias the results and therefore the number of missing data should be reported. The percentage of missing data per indicator is preferably less than 10%.

The missing data of the indicators used in this study were between 0.7%-3.1%.

- ***Discriminatory capacity:*** the discriminatory capacity indicates whether an indicator can discriminate practice performance between different cancer centers. Sometimes indicators are measured for which a large part of cancer centers will have similar scores. These indicators with invariable scores will not be able to discriminate between care providers or cancer centers and therefore contain less information. High discriminatory capacity is therefore present when the range in scores between the lowest- and highest-scoring cancer center is >20%[^7^](#_ENREF_7).

The range of percentage of adherence for screening with the DT, information provision about physical activity (PA) and physical cancer rehabilitation programs (PCRPs), referral to PCRPs and participation in PCRPs was >20%. The range was <20% for the indicators advice to take part in PA and PCRPs and PAU.

- ***Complexity:*** the concept of complexity was used for the number of variables needed to fill the numerator and denominator of an indicator. Complexity is thus a measure that reflects the amount of investments needed to assess an indicator; the higher the complexity the more effort it takes to measure an indicator.

The complexity of the indicators used in this study was low. One variable was needed per indicator to measure the indicator[^9^](#_ENREF_9).

**References**

1. Hermens RP, Ouwens MM, Vonk-Okhuijsen SY, et al. Development of quality indicators for diagnosis and treatment of patients with non-small cell lung cancer: a first step toward implementing a multidisciplinary, evidence-based guideline. *Lung Cancer.* Oct 2006;54(1):117-124.

2. Mourad SM, Hermens RP, Nelen WL, Braat DD, Grol RP, Kremer JA. Guideline-based development of quality indicators for subfertility care. *Hum Reprod.* Oct 2007;22(10):2665-2672.

3. Fitch K, Bernstein SJ, Dolores Aguilar M, et al. *The RAND/UCLA Appropriateness Method User's Manual.* 2001.

4. Kotter T, Blozik E, Scherer M. Methods for the guideline-based development of quality indicators--a systematic review. *Implement Sci.* Mar 21 2012;7:21.

5. McGlynn EA. Selecting common measures of quality and system performance. *Med Care.* Jan 2003;41(1 Suppl):I39-47.

6. Rubin HR, Pronovost P, Diette GB. From a process of care to a measure: the development and testing of a quality indicator. *Int J Qual Health Care.* Dec 2001;13(6):489-496.

7. Grol R, Baker R, Moss F. Quality improvement research: understanding the science of change in health care. *Qual Saf Health Care.* Jun 2002;11(2):110-111.

8. Wennekes L, Ottevanger PB, Raemaekers JM, et al. Development and measurement of guideline-based indicators for patients with non-Hodgkin's lymphoma. *J Clin Oncol.* Apr 10 2011;29(11):1436-1444.

9. Mourad SM, Nelen WL, Hermens RP, et al. Variation in subfertility care measured by guideline-based performance indicators. *Hum Reprod.* Nov 2008;23(11):2493-2500.
