# Supplementary material for: Determinants of adherence to physical cancer rehabilitation guidelines among cancer patients and cancer centers: a cross-sectional observational study
Source: J Cancer Surviv. 2020 Sep 28;15(1):163–77. doi: 10.1007/s11764-020-00921-8 (PMC7822788; doi:10.1007/s11764-020-00921-8)
Supplement: Supplementary file 2 — (DOCX 18.7 kb). [file 11764_2020_921_MOESM2_ESM.docx]

**Supplement 2 overview of the range of potential values of each developed and measured indicator regarding the psychometric characteristics**

| **No.** | **Indicator** | **Validity** | **Reliability** | **Measurability** | **Applicability** | **Improvement**  **Potential** | **Missing data** | **Discriminatory capacity at hospital level** | **Complexity** |
| --- | --- | --- | --- | --- | --- | --- | --- | --- | --- |
|  |  |  |  |  | >10%= Yes | <90% adherence = Yes | <10%= Yes | >20%=Yes | <5 variables=Yes |
|  |  |  |  |  | Number of patients (%*) | Number of patients (%*) | Number of patients with missing data (%*) | Range %-%* (difference%*) | (N-variables) |
| *1* | Screening with the Distress Thermometer | *Yes* | *Yes* | *Yes* | *999 (100.0)* | 468 (47.2) | *7 (0.7)* | 29.5%-60.1% (30.6%) | 1 |
| *2* | Information provision concerning PA and PCRPs | *Yes* | *Yes* | *Yes* | *999 (100.0)* | 427 (44.1) | *31 (3.1)* | 30.8%-54.6% (23.8%) | 1 |
| *3* | Advice to take part in PA and PCRPs | *Yes* | *Yes* | *Yes* | *999 (100.0)* | 550 (55.6) | *10 (1.0)* | 48.0%-65.0% (17%) | 1 |
| *4* | Referral to PCRPs | *Yes* | *Yes* | *Yes* | *999 (100.0)* | 174 (17.7) | *17 (1.0)* | 3.7%-31.0% (27.3%5) | 1 |
| *5* | Participation in PCRPs | *Yes* | *Yes* | *Yes* | *999 (100.0)* | 280 (28.6) | *21 (2.1)* | 18.6%-41.5% (22.9%) | 1 |
| *6* | Patient PA uptake (PAU) | *Yes* | *Yes* | *Yes* | *999 (100.0)* | 446 (45.3) | *15 (1.5)* | 41.3%-52.5% (11.2%) | 1 |

Abbreviations: No, number; PA, physical activity; PCRP: Physical Cancer Rehabilitation Program; PAU, physical activity uptake

*valid percentage
