# Supplementary material for: Determinants of adherence to physical cancer rehabilitation guidelines among cancer patients and cancer centers: a cross-sectional observational study
Source: J Cancer Surviv. 2020 Sep 28;15(1):163–77. doi: 10.1007/s11764-020-00921-8 (PMC7822788; doi:10.1007/s11764-020-00921-8)
Supplement: Supplementary file 3 — (DOCX 30.8 kb). [file 11764_2020_921_MOESM3_ESM.docx]

**Supplement 3 Description of the questionnaires used**

***Distress Thermometer (DT)***[^1^](#_ENREF_1)***:*** The DT consists of a thermometer ranging from 0 (no distress) to 10 (extreme distress) and 47 questions (yes or no) that refer to practical, family and social, emotional, religious and spiritual, and physical issues. The DT concludes with the question: “Would you like to talk with a professional about your problems?” (yes, no, or maybe). The cut-off point is 5. The DT showed valid and reliable. The sensitivity of the DT is 78.3%, whereas its specificity is 66.5%[^1^](#_ENREF_1).

***The European Organization for Research and Treatment of Cancer Quality of Life Questionnaire (EORTC QLQ-C30)***[***^2^***](#_ENREF_2)***:*** This self-completion questionnaire consists of 30 questions and is composed of multi-item and single scales. There are five functional scales (physical, role, emotional, social, and cognitive), three symptom scales (fatigue, nausea or vomiting, and pain), a global health status or quality of life scale, and six single items (dyspnea, insomnia, appetite loss, constipation, diarrhea, and financial difficulties). A measurement model for the QLQ-C30 that yields a single summary score based on 13 scales (27 items) was also calculated[^3^](#_ENREF_3). The QLQ-C30 and the single summary score both showed valid and reliable[*^2^*](#_ENREF_2)*^,^*[*^3^*](#_ENREF_3).

***The Multidimensional Fatigue Inventory-20 (MFI-20) Questionnaire***[***^4^***](#_ENREF_4)***^,^***[***^5^***](#_ENREF_5)***:*** The MFI-20 is a 20-item scale designed to evaluate five dimensions of fatigue: general fatigue, physical fatigue, reduced motivation, reduced activity, and mental fatigue. The MFI-20 showed reliable and valid to differentiate fatigue between groups, within groups and for patients with cancer[*^4^*](#_ENREF_4)*^,^*[*^5^*](#_ENREF_5)*.*

***The Patient ActivityMeasurement-13 (PAM-13)***[***^6^***](#_ENREF_6)***^,^***[***^7^***](#_ENREF_7)**:** The PAM-13 is the shortened 13-item version of the PAM-22, a 22-item measure that assesses patient knowledge, skill, and confidence for self-management. The PAM-13 divides people into one of four progressively higher activation levels, from passive and lacking knowledge and skills in dealing with health and healthcare in level 1 to active and generally well-informed and competent in level 4. The original PAM-22 showed valid and reliable to measure the level of activation and the shorter PAM-13 showed valid compared to the original 22-item PAM-22[*^6^*](#_ENREF_6)*^,^*[*^7^*](#_ENREF_7)*.*

**References**

1. Tuinman MA, Gazendam-Donofrio SM, Hoekstra-Weebers JE. Screening and referral for psychosocial distress in oncologic practice: use of the Distress Thermometer. *Cancer.* Aug 15 2008;113(4):870-878.

2. Aaronson NK, Ahmedzai S, Bergman B, et al. The European Organization for Research and Treatment of Cancer QLQ-C30: a quality-of-life instrument for use in international clinical trials in oncology. *J Natl Cancer Inst.* Mar 3 1993;85(5):365-376.

3. Giesinger JM, Kieffer JM, Fayers PM, et al. Replication and validation of higher order models demonstrated that a summary score for the EORTC QLQ-C30 is robust. *J Clin Epidemiol.* Jan 2016;69:79-88.

4. Smets EM, Garssen B, Cull A, de Haes JC. Application of the multidimensional fatigue inventory (MFI-20) in cancer patients receiving radiotherapy. *Br J Cancer.* Jan 1996;73(2):241-245.

5. Smets EM, Garssen B, Bonke B, De Haes JC. The Multidimensional Fatigue Inventory (MFI) psychometric qualities of an instrument to assess fatigue. *J Psychosom Res.* Apr 1995;39(3):315-325.

6. Hibbard JH, Stockard J, Mahoney ER, Tusler M. Development of the Patient Activation Measure (PAM): conceptualizing and measuring activation in patients and consumers. *Health Serv Res.* Aug 2004;39(4 Pt 1):1005-1026.

7. Hibbard JH, Mahoney ER, Stockard J, Tusler M. Development and testing of a short form of the patient activation measure. *Health Serv Res.* Dec 2005;40(6 Pt 1):1918-1930.
